# Macromolecular *ab initio* phasing enforcing secondary and tertiary structure

**DOI:** 10.1107/S2052252514024117

**Published:** 2015-01-01

**Authors:** Claudia Millán, Massimo Sammito, Isabel Usón

**Affiliations:** a Structural Biology, Molecular Biology Institute of Barcelona, Baldiri Reixac 15, Barcelona, 08028, Spain; b Structural Biology, ICREA at IBMB-CSIC, Baldiri Reixac 13-15, Barcelona, 08028, Spain

**Keywords:** *ab initio* phasing, α-helices, macromolecular structure, *ARCIMBOLDO*

## Abstract

*Ab initio* phasing of macromolecular structures, from the native intensities alone with no experimental phase information or previous particular structural knowledge, has been the object of a long quest, limited by two main barriers: structure size and resolution of the data. Current approaches to extend the scope of *ab initio* phasing include use of the Patterson function, density modification and data extrapolation. The authors’ approach relies on the combination of locating model fragments such as polyalanine α-helices with the program *PHASER* and density modification with the program *SHELXE*. Given the difficulties in discriminating correct small substructures, many putative groups of fragments have to be tested in parallel; thus calculations are performed in a grid or supercomputer. The method has been named after the Italian painter Arcimboldo, who used to compose portraits out of fruit and vegetables. With *ARCIMBOLDO*, most collections of fragments remain a ‘still-life’, but some are correct enough for density modification and main-chain tracing to reveal the protein’s true portrait. Beyond α-helices, other fragments can be exploited in an analogous way: libraries of helices with modelled side chains, β-strands, predictable fragments such as DNA-binding folds or fragments selected from distant homologues up to libraries of small local folds that are used to enforce nonspecific tertiary structure; thus restoring the *ab initio* nature of the method. Using these methods, a number of unknown macromolecules with a few thousand atoms and resolutions around 2 Å have been solved. In the 2014 release, use of the program has been simplified. The software mediates the use of massive computing to automate the grid access required in difficult cases but may also run on a single multicore workstation (http://chango.ibmb.csic.es/ARCIMBOLDO_LITE) to solve straightforward cases.

## Abstract 2

*ARCIMBOLDO* replaces the atomicity constraints required for *ab initio* phasing by enforcement of model stereochemistry. Small model fragments and local folds are exploited at resolutions up to 2 Å in different contexts, from supercomputers to the standalone *ARCIMBOLDO_LITE*, which solves straightforward cases on a single multicore machine.

## 1. Introduction   

**Figure 1:**
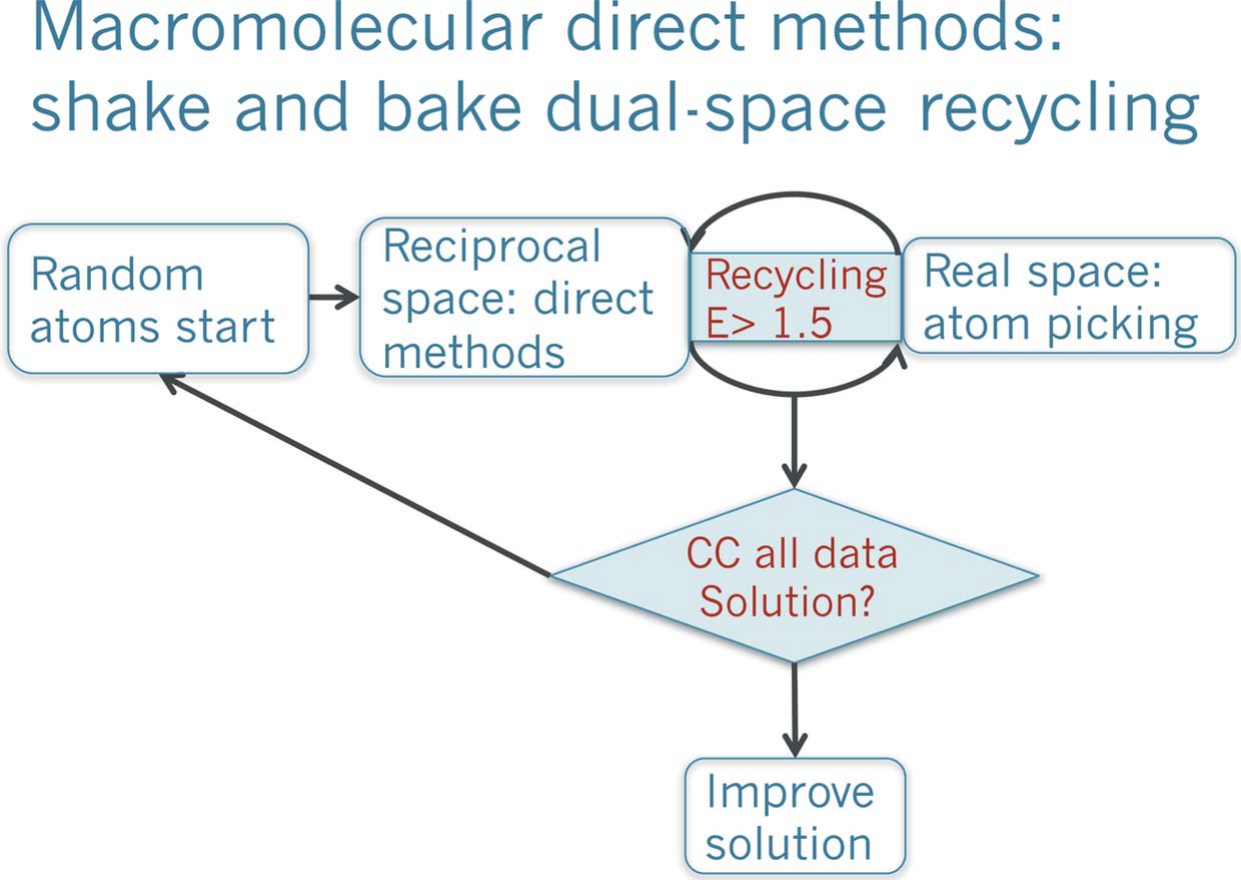
Dual-space recycling *Shake-and-Bake* algorithm for *ab initio* phasing at atomic resolution.

One hundred years have passed since Max von Laue was awarded the Nobel Prize in Physics for his discovery of the diffraction of X-rays by crystals (Friedrich *et al.*, 1912 ▶; von Laue, 1912 ▶). Since that discovery, crystallography has become an essential tool of investigation throughout the sciences, as it provides information on molecular structure down to the atomic level with a degree of detail and accuracy that is unsurpassed by any other structural technique. X-ray diffraction was first used by the Braggs to determine the three-dimensional structure of crystals (Bragg & Bragg, 1913 ▶). In a diffraction experiment only the intensities of the diffracted X-ray beams are recorded, whereas their phases are not. Nevertheless, phases are required to compute an electron-density map from which an atomic model can be derived. Providing the missing phases has been a quest since the beginning of crystallography and phasing still constitutes a bottleneck in many crystallographic studies. In the field of macromolecular crystallography, initial phases are usually derived either experimentally from a substructure of reference atoms, intrinsic to the structure or incorporated, and data collected at one or more particular wavelengths (Hendrickson, 1991 ▶), or from the placement in the asymmetric unit of a model related to the target structure (Rossmann, 1972 ▶). In chemical crystallography, for structures composed of fewer than 200 independent atoms, direct methods (Hauptman & Karle, 1953 ▶; Karle & Hauptman, 1956 ▶) are generally able to provide an initial model exclusively from the experimental intensities measured on a native crystal. Unlike in macromolecular crystallography, no previous stereochemical knowledge or additional experimental data from modified crystals or selected wavelengths are needed. Direct methods are therefore termed *ab initio* methods. They solve the phase problem exploiting probabilistic relations and the possibility of evaluating many starting phase sets through reliable figures of merit. The extension of direct methods to larger structures of around 1000 independent atoms was accomplished by the introduction of the *Shake-and-Bake* algorithm (Miller *et al.*, 1993 ▶) implemented in the programs *SnB* (Miller *et al.*, 1994 ▶) and *SHELXD* (Usón & Sheldrick, 1999 ▶). Fig. 1 ▶ shows a scheme of the *Shake-and-Bake* algorithm (Sheldrick *et al.*, 2011 ▶). Starting from an initial hypothesis, usually a set of randomly generated atoms, phases are calculated and modified according to direct methods relationships. The modified phases are used to calculate an electron-density map and a new set of atoms is selected from the maxima in this map. In favourable cases, iteration of this process leads to a structure solution, which can be identified by a reliable figure of merit called the correlation coefficient (CC) (Fujinaga & Read, 1987 ▶). It should be noted that all steps in the procedure described enforce atomicity as a constraint: the initial phase set is calculated from a (random) atomic model, the tangent formula and minimal function are derived from atomicity and the calculated maps are interpreted by picking atoms from which to calculate a new set of phases. It is therefore not surprising that such methods were limited by the requirement of atomic resolution data. Table 1 ▶ summarizes the previously unknown structures with more than 300 independent atoms which were solved *ab initio* using *SHELXD*. Remarkably, the table features a large number of nonstandard macromolecules, such as antibiotics or large disulfide-rich peptides for which classic protein methods did not provide an adequate alternative as neither suitable models nor easy ways of derivatization were an option. For example, the structure of the antibiotic vancomycin had long been awaited, as its crystallization had been described many years before a solution was independently achieved with *SHELXD* (Schäfer *et al.*, 1996 ▶) and *SnB* (Loll *et al.*, 1997 ▶).

**Figure 2:**
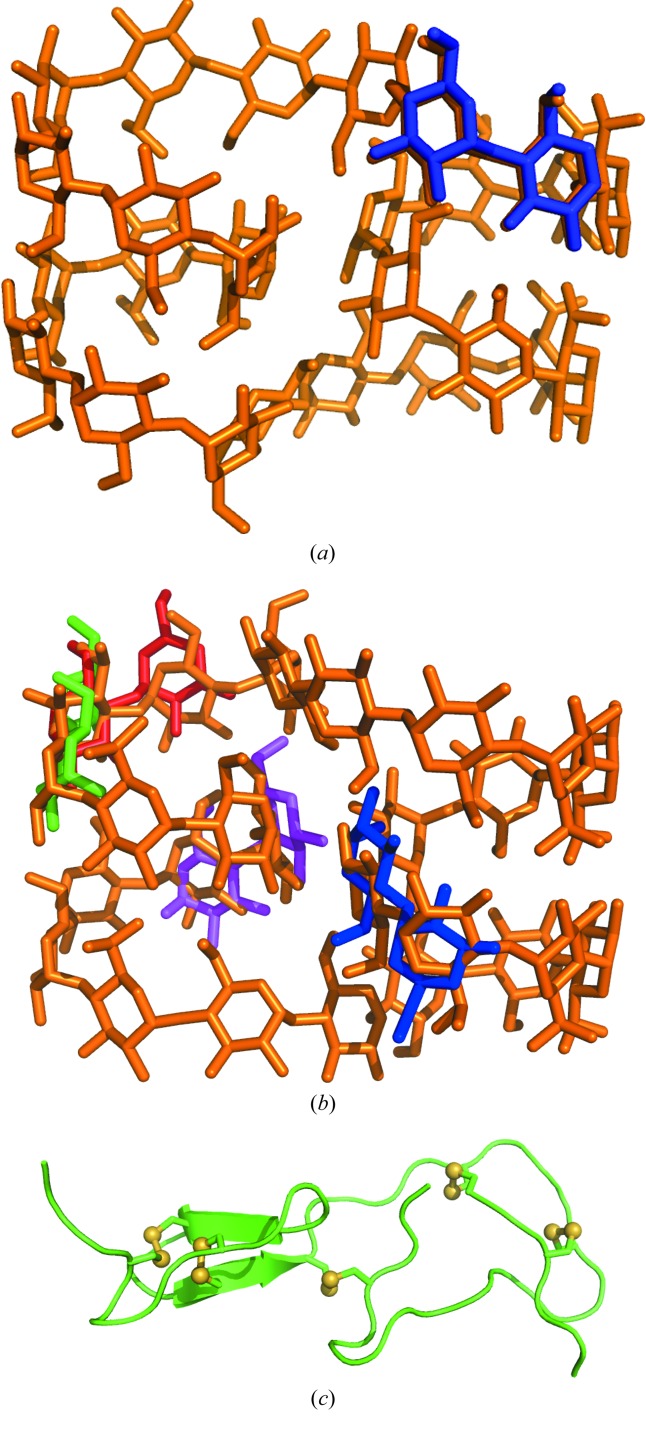
Structures of the cycloamylose CA26 (cyclomaltohexaicosaose) in space groups (*a*) *P*1 and (*b*) *P*2_1_2_1_2_1_. These structures were solved starting from a randomly placed and locally optimized diglucose fragment. Different start locations led to the same final solution. (*c*) Structure of hirustasin, solved locating first the substructure made from the ten sulfur atoms in the five disulfide bridges and expanding from that point to the whole structure.

Macromolecular structures diffracting to atomic resolution (1.2 Å or beyond) are rather an exception [less than 2.5% of the entries in the PDB (Bernstein *et al.*, 1977 ▶; Berman *et al.*, 2000 ▶)]. A general *ab initio* phasing method should also be able to tackle lower resolution cases. Still, a number of useful experiences can be drawn from the atomic resolution cases and exported to the lower resolution application. Some of the structures solved *ab initio* with *SHELXD* required the location of a small fragment of known geometry to generate the initial hypothesis, rather than relying on a collection of totally random atoms. For instance, the cycloamylose CA26, in the triclinic *P*1 form (Gessler *et al.*, 1999 ▶) or in the orthorhombic *P*2_1_2_1_2_1_ form (Nimz *et al.*, 2004 ▶), displayed in Figs. 2 ▶(*a*) and 2(*b*) respectively, could only be solved by locally optimizing the random positioning of a diglucose fragment to seed the *Shake-and-Bake* procedure. Similarly, the structure of hiru­stasin (Usón *et al.*, 1999 ▶), shown in Fig. 2 ▶(*c*), could be solved not only from the original 1.2 Å data, but even from a 1.4 Å data set by initially locating the substructure made up by the ten sulfur atoms in its five disulfide bridges at the stage where the algorithm works on the strongest normalized data and extending this substructure through iterative map interpretation against all data. Benchmarks on test structures showed that a large number of cycles could lead to a solution even switching off the direct methods part of the algorithm, that is with no modification at the reciprocal-space stage (Sheldrick *et al.*, 2011 ▶). Indeed, plain recycling of the map interpretation stage, through atom picking and randomly omitting one third of them, was able to solve the 317 atom test structure of gramicidin A (Langs, 1989 ▶).

In the dual-space recycling methods, not every attempt to phase a given structure results in a solution. *SHELXD* pursues many start hypotheses and keeps the so far best result, for particularly difficult cases many trials may be required to produce one successful solution, for instance it took 25 000 starting sets of atoms to achieve one solution of polyA RNA (Safaee *et al.*, 2013 ▶). The multisolution approach requires an effective way of identifying successful solutions or the ones that are susceptible to being improved, as it is not possible to examine all resulting electron-density maps or atomic models. The correlation coefficient (CC) calculated on all data is reliable when atomic resolution data are available but at lower resolution, all random collections of a large enough number of unconstrained atoms show equally high CC values. Atomic resolution and completeness of the data were also found to be essential for phasing with the program *SnB* (Xu *et al.*, 2000 ▶). Extrapolation to include the non-measured data was introduced by Giacovazzo (Caliandro *et al.*, 2005*a* ▶) to improve the experimental data when these conditions were not fulfilled and its use was incorporated into the *ab initio* phasing case (Caliandro *et al.*, 2005*b* ▶). The presence of heavier atoms than sulfur, in the form of inherent metals or counter ions, is also beneficial leading to larger structures being solved, such as a cytochrome c3 with 2208 atoms, including eight Fe atoms (Frazao *et al.*, 1999 ▶). This advantage has been exploited in *ab initio* phasing through sophisticated use of the Patterson function (Caliandro *et al.*, 2008 ▶).

Approximately half (48%) of the deposited crystal structures in the PDB diffract to 2 Å or better. Therefore, an *ab initio* phasing method effective up to such resolution would be of general interest. The approach underlying the *ARCIMBOLDO* method is structured around the following ideas. To break the atomic resolution dependency, it should be instrumental to substitute the enforcement of atomicity by that of stereochemical knowledge of larger units. In practice, phasing should be constrained by fragments, rather than by atoms. Also, instead of atom picking to interpret a map, density modification would produce effective improvement at lower resolution. Giacovazzo *et al*. have extensively developed this aspect in their VLD algorithm (Burla *et al.*, 2011 ▶, 2012 ▶). Starting the phasing from a small but highly accurate substructure in the context of *ACORN* was reported to be remarkably effective (Yao *et al.*, 2005 ▶, 2006 ▶) and our own tests corroborated this finding, as little as 10% of the main-chain atoms suffice to solve a structure at 2 Å. Again, automatic interpretation of the modified maps in the form of peptide main-chain tracing constrains phasing towards the correct solution and at the same time provides a reliable figure of merit. Whereas the CC for an unrestrained collection of atoms may also be high for an incorrect substructure, a wrong trace can be clearly discriminated from a correct one with sensible stereochemistry at resolutions up to 2.0 Å. Up to this point, where autotracing identifies a solution, it may not be possible to discriminate partially correct from wrong solutions. This imposes the need to compute many different hypotheses and to develop them to a stage where success can be identified, entailing a large demand on CPU time. The process is easy to parallelize and task distribution on a large grid or supercomputer has been an essential element to this method.

The present work deals with approaches to phase structures *ab initio* substituting the need for atomic resolution by stereochemical knowledge through secondary structure fragments and local folds.

## 2. *ARCIMBOLDO*   

**Figure 3:**
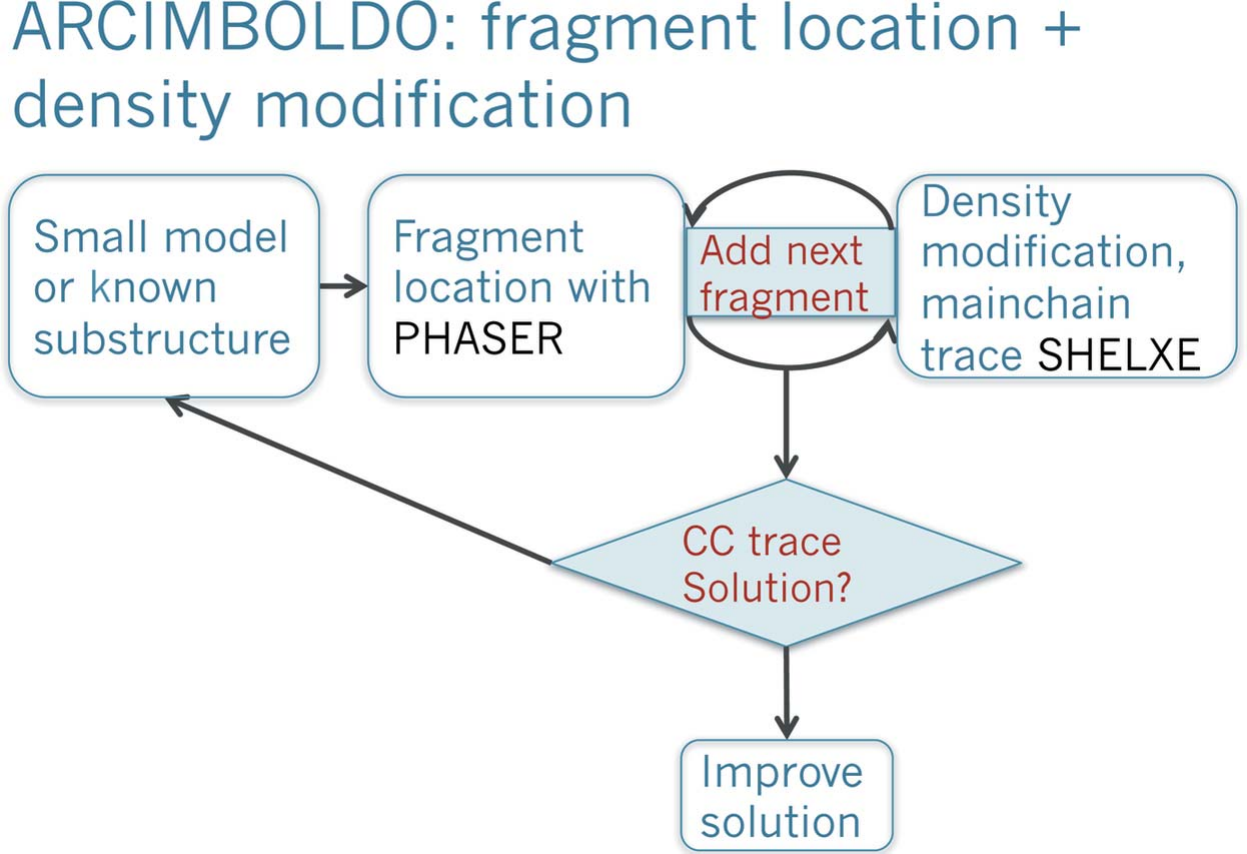
*ARCIMBOLDO* algorithm for *ab initio* phasing with model fragments at resolution up to 2 Å.

Building on the atomic resolution dual-space recycling experience, the central idea in our approach to overcome the resolution barrier and to extend the scope of *ab initio* phasing to resolutions up to 2 Å was to substitute atomicity constraints by the enforcement of a secondary structure. Rather than starting the phasing from a collection of atoms, secondary structure model fragments would be randomly placed and their starting position locally optimized or alternatively located with the program *PHASER* (McCoy *et al.*, 2007 ▶). Instead of improving phases through the tangent formula and interpreting as atoms the maxima in the electron-density maps produced, maps would be improved by density modification techniques and the improved maps would be interpreted in terms of the main chain with the program *SHELXE* (Sheldrick, 2002 ▶). Main chain autotracing would in turn provide a reliable figure of merit at the proposed resolution (Sheldrick, 2010 ▶). The CC characterizing the trace is distinctly higher for correct rather than for wrong traces (Thorn & Sheldrick, 2013 ▶). Fig. 3 ▶ displays a scheme of this approach. We named the method after the 16th century painter Arcimboldo, who assembled portraits out of objects such as fruit and vegetables. Our starting hypothesis assembles partial structures out of secondary structure fragments and, if correct enough, density modification succeeds in revealing the portrait of our protein, expanding to a nearly complete structure. As most of our trials remain a ‘still life’, the method requires extensive computing. Fortunately, the calculations can be easily split into small tasks and distributed over a grid of computers or a supercomputer.

### 2.1. α-Helices as ideal fragments   

**Figure 4:**
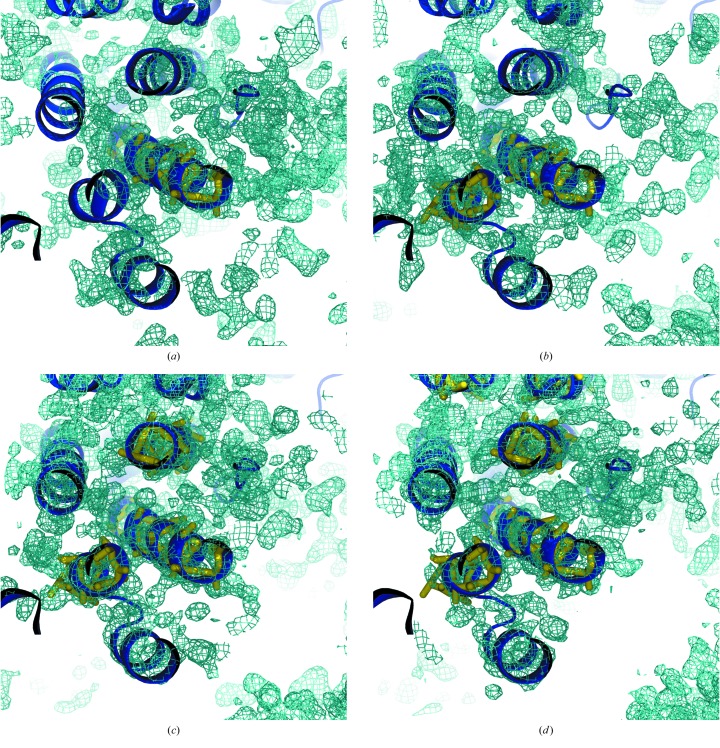
Stepwise evolution in the solution of myosin Vb (PDB ID 4j5m). The four panels display the *Dm* *F*_o_ − *F*_c_ maps contoured at 1σ calculated after placement of successive helices of 22 alanines and at the final stage. The initial mean phase errors, CC for the starting substructure and number of residues traced are quoted for each panel. (*a*) After placement of one helix of 22 amino acids, initial CC is 7.21% and wMPE is 73.4°, 64 residues; (*b*) after placement of two helices of 22 amino acids, initial CC is 10.0% and wMPE is 68.0°, 76 residues; (*c*) after placement of three helices of 22 amino acids, initial CC is 12.7% and wMPE is 62.7°, 127 residues; (*d*) final solution, initial CC is 26.6% and wMPE is 53.4°, 241 residues traced and final wMPE is 42°. The figure was prepared with *PyMOL*.

The obvious ideal fragment to start from was that composed of the main chain atoms of a regular α-helix. They have been used for phasing in a stochastic multidimensional search, representing less than 13% of the total number of atoms per fragment (Glykos & Kokkinidis, 2003 ▶). α-Helices are nearly ubiquitous as 80% of the structures deposited in the PDB contain at least one of more than 12 residues. They are also constant in their geometry, so that a helix of 14–16 residues will fit to the main chain of almost any helix on any structure with an r.m.s.d. below 0.3 Å. Rather rigid, helices will often have low-*B* values in relation to the rest of the structure. Finally, we did not anticipate it but we probably get an added benefit from their being periodic, which gives rise to characteristic features in the Patterson function (Caliandro *et al.*, 2012 ▶). The first unknown structure solved by *ARCIMBOLDO*, was that of the PRD2, containing 220 amino acids in the asymmetric unit and diffracting to a resolution of 1.95 Å (Rodríguez *et al.*, 2009 ▶). A solution was reached in the case of three out of the 1467 partial structures combining three main-chain helices of 14 alanines. Since then, as can be seen in Table 2 ▶, at least 18 new structures have been solved from helices. From the first ‘brute-force’ implementation that generated large numbers of partial structures and attempted expansion of as many of them as possible for a given setup of computing resources, examination of intermediate results has allowed a more rational control of the process. Fig. 4 ▶ illustrates the stages in the solution of myosin Vb at 2.07 Å (Nascimento *et al.*, 2013 ▶). Fig. 4 ▶(*a*) displays the *SHELXE* electron-density map resulting after placement of a single helix, density modification and autotracing. Besides the correctly placed helix displayed, the still very noisy map, characterized by a mean phase error (MPE) of 73°, shows electron density around some places where missing helices should be. Fig. 4 ▶(*b*), after placement of a second helix, displays a more correct map with an MPE of 68°, where correct features in the structure start to emerge but do not develop into a full structure. Fig. 4 ▶(*c*), after placement of a third helix produces an initial map of 63° MPE whose recycling, shown in Fig. 4 ▶(*d*), leads to more than two thirds of the main chain being built and a final map of 42° MPE, where electron density for some of the side chains also becomes apparent.

#### 2.1.1. Rotation of helices   

**Figure 5:**
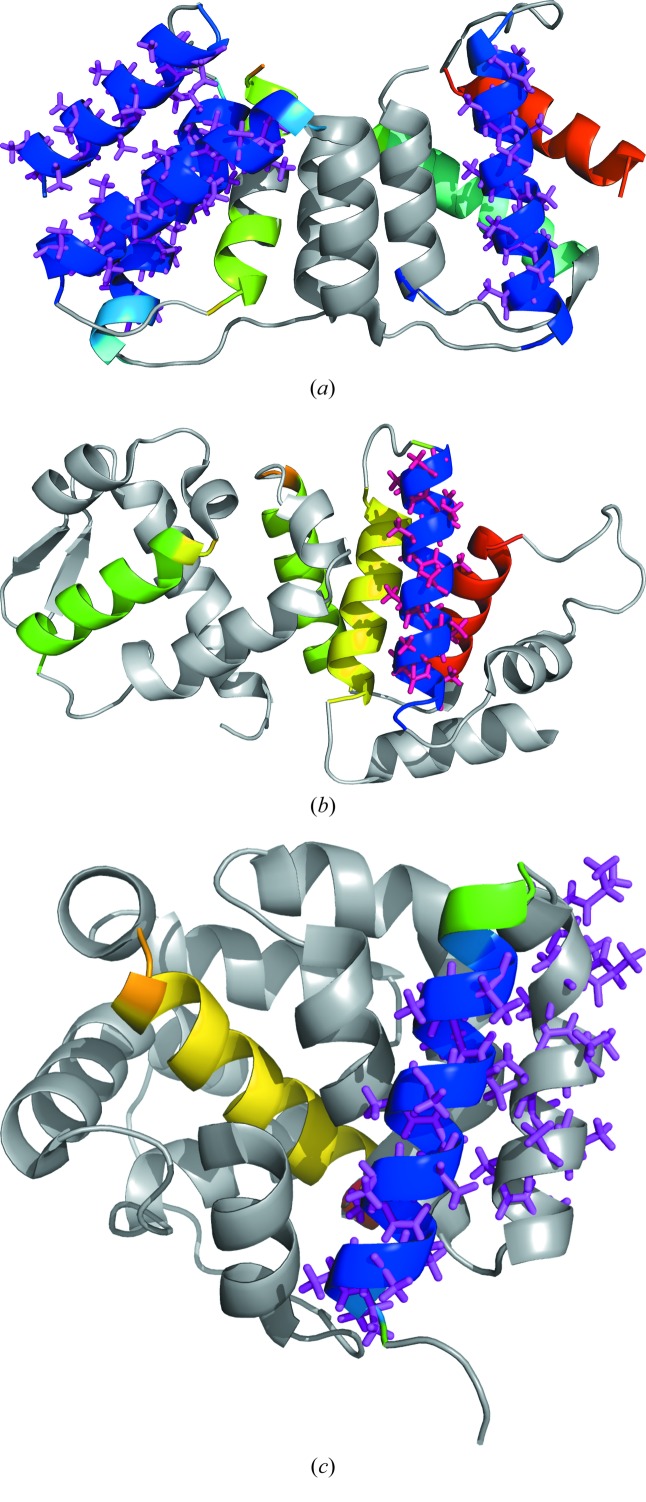
Structures of (*a*) PRD2 (PDB ID 3gwh), (*b*) *Lv*-ranaspumin (4k82) and (*c*) eIF5 (2iu1) in cartoon representation. Helices of 14 amino acids or more are coded with a rainbow scheme to represent the LLG value of the rotation function characterizing each of the possible helices that can be fitted. Blue indicates a high LLG value and red a low one. Helices whose rotation was found in a search at full resolution (threshold, 75% of top) are represented as magenta sticks. The figure was prepared with *Coot* and *PyMOL*.

Given an all-helical protein, it is interesting to examine whether or not all helices are represented in the partial solutions, how independent solutions are at the first stage of a *PHASER* rotation search (Storoni *et al.*, 2004 ▶) and how they develop as the search for additional fragments proceeds. Let us consider a few representative cases, displayed in Fig. 5 ▶. For the protein PRD2 (PDB ID 3gwh) described above, containing 220 amino acids involving ten helical stretches of lengths ranging from 10 to 20 amino acids, diffraction data to 1.95 Å are available. A full resolution rotation search produces 42 solutions within 75% of the top log-likelihood gain (LLG) value. They can be clustered within a tolerance of 15° into six independent rotations, taking into account the space group symmetry. Four of the six clusters correspond to helices in the structure, the remaining two have errors such as mapping to the rotation representing a smaller helix than the search fragment or large deviations from the geometrically closest rotation corresponding to a helix in the true structure. Fig. 5 ▶(*a*) displays the PRD2 structure, representing the four correctly identified rotations superimposed on their corresponding correct locations. In this structure, 41 overlapping stretches are compatible with a model helix 14 amino acids long, with an r.m.s.d. ranging from 0.29 to 0.36 Å. Calculation of the LLG values with the MR_GYRE mode in *PHASER* allows us to rank those helices that have the best rotation function scores and could therefore be located. Results have been coded with rainbow colours, blue representing the highest and red the lowest LLG values. As can be seen in Fig. 5 ▶(*a*), three of the possible helices in the structure present much lower rotation LLG values, their location being highly improbable as their LLG values fall outside the 75% limit.

The protein eIF5 (Bieniossek *et al.*, 2006 ▶) (PDB ID 2iu1), displayed in Fig. 5 ▶(*b*), contains 179 amino acids in 11 helical stretches of lengths ranging from seven to 21 amino acids and for which diffraction data to 1.7 Å are available. A rotation search at full resolution yields 25 peaks within 75% of the top LLG value, which can be grouped into four clusters, two of them match true helices with an r.m.s.d. below 0.3 Å.

For *Lv*-ranaspumin (Hissa *et al.*, 2014 ▶) (PDB ID 4k82) at 1.7 Å, all 26 rotation peaks correspond to the same correct helix in the final structure, a second cluster shows an r.m.s.d. of 1.26 Å to the closest real helix.

In summary, not all helices in a structure are equally represented in a rotation search, even reducing the sampling step. Long helices with lower *B* values especially appear to be more successfully located.

#### 2.1.2. Translation of helices   

A translation search (McCoy *et al.*, 2005 ▶) requires highly accurate rotations to succeed, but is sometimes unexpectedly able to accommodate errors such as the displacement of a helix by one or more residues falling outside the correctly placed helix or a high local deviation as long as the core of the placed model is very accurate. In the three cases described, the correct translation is located for half of the helices where a correct rotation was recognisable, in particular those cases where the rotation was most accurate.

#### 2.1.3. Helices with side chains   

Any model(s) in PDB format can be specified as a search fragment. Our first implementation (Rodríguez *et al.*, 2012 ▶) contemplated evaluating libraries of alternative models against the rotation function and proceeding on with the best scoring in terms of rotation LLG or *Z* score. Tests showed that it was possible to select the helix with side chains set to closest conformers to aid phasing by using more complete models. The use of libraries has moved in the current implementation to the more sophisticated treatment in the *ARCIMBOLDO_BORGES* mode explained below. Helices with side chains as fragments were successfully used in the first solution of a muscle atrogin coiled-coil structure (Franke *et al.*, 2014 ▶) but the current version succeeds from polyalanine fragments as well.

### 2.2. β-Strands   

The geometry of β-strands is inherently more varied, as can be appreciated from a glance at a typical Ramachandran plot (Ramachandran *et al.*, 1963 ▶). All amino acids in α-helices are found within a very narrow range of ϕ, ψ angles around the preferred −63.8°, −41.1° region. As many as 40% of all amino acids are found in this most populated region, covering only 2% of the Ramachandran plot. The β-sheet region is clearly subdivided into two distinct regions and the standard deviations around the two maxima are as high as 20° for nonproline and nonglycine amino acids (Hovmöller *et al.*, 2002 ▶).

The structure of the dimeric colicin immunity protein CMI (Usón *et al.*, 2012 ▶) from *Escherichia coli* contains 115 amino acids in the asymmetric unit and its fold displays a sheet of four antiparallel β-strands and three helices, the longest one comprising 26 residues. Diffraction data in the space group *C*222_1_ are available to a resolution of 1.8 Å. This small protein was used as a test to try to solve it from either a helical model or from an equivalent β-strand. Searching for a nine amino-acid-long model polyalanine helix solves the structure, while it must be noted that typical search helices are usually longer. In contrast, not even the longest strand in the structure spanning nine residues (from 97 to 105) could be successfully used, even including side chains in their true conformation. Such a perfect model is impossible to predict and thus, isolated model strands are of limited use. Despite the higher variability in strand conformation, their association into a sheet fold tends to be more constant, as it involves main-chain hydrogen bonds rather than the side-chain mediated contacts linking neighbour helices. The structure can be solved from a double-stranded perfect model, indicating that small local folds should provide a better search model for β-sheets than relying on isolated strands.

### 2.3. DNA-binding fragments   

Small local folds may be predicted with enough accuracy to provide suitable models. Clearly, such an approach involves the use of previous structural knowledge particular to the macromolecule to be phased and cannot be considered an *ab initio* method, unless the DNA helix suffices as a search fragment in an analogous role to that of the α-helix. RNA secondary structure elements have been used as multiple search fragments in an effective method, combining molecular replacement (MR), manual map inspection, refinement, density modification and composite-omit maps (Robertson & Scott, 2008 ▶; Robertson *et al.*, 2010 ▶). In order to enable structure solution with *ARCIMBOLDO*, we have suggested taking advantage of the specific patterns of DNA-binding proteins to generate databases of conserved structural motifs (Pröpper *et al.*, 2014 ▶). Precomputed libraries can be downloaded from our web site (http://chango.ibmb.csic.es/dna) or calculated on the fly to structurally resemble an input PDB template.

## 3. *SHREDDER*   

In an analogous way, if a remote homologue is known but MR fails, it frequently occurs that part of the target structure will resemble the search model in its fold. Sophisticated methods exist to predict from sequence and structure statistics how to trim and modify such a template to produce MR search models These involve approaches for model weighting, enhancement and combination such as the ones found in *Sculptor* (Bunkóczi & Read, 2011 ▶), *mrtailor* (Gruene, 2013 ▶), *SCEDS* (McCoy *et al.*, 2013 ▶) or *Ensembler* (Bunkóczi *et al.*, 2013 ▶). A potential approach would be to extract all sorts of possible small fragments from the model of the distant homologue and use them as search models within *ARCIMBOLDO*. The number of reasonable structural hypotheses becomes very large and therefore the algorithm in *ARCIMBOLDO_SHREDDER* was designed to select the best search models by optimization against the experimental diffraction data, rather than on expectations based on previous knowledge. Evaluation of each residue in the template is carried out through analysis of the Shred-LLG function, combining the LLG results of a number of rotation functions (Storoni *et al.*, 2004 ▶) calculated on systematically shredded models (Sammito *et al.*, 2014 ▶). A few models resulting from omitting all less suitable spans, as indicated by the Shred-LLG function values, are used as *ARCIMBOLDO* search fragments.

## 4. *BORGES*   

In the absence of specific knowledge, we would expect that any unknown structure should contain local folds already seen in the PDB, but how would we retrieve and exploit this information? Our program *BORGES* was developed to identify, retrieve and exploit unspecific tertiary structure through libraries of fragments (Sammito *et al.*, 2013 ▶). The PDB database contains a vast amount of information and for any unknown structure, given small enough fragments, such as the main chain of two helices or three strands in a particular disposition, similar models to an accuracy bordering 0.5 Å r.m.s.d. are bound to occur in some of the deposited entries. In analogy to Borges’ infinite ‘Library of Babel’ that contained books with all random combinations of letters and therefore enclosed any possible book, the partial models required to phase a structure through fragment search and density modification should already have been described within other structures deposited in the PDB. Unlike the ‘Borges library’, the PDB is non-random, containing in all sorts of structural contexts only meaningful structural units. In addition, our phasing method requires small sentences rather than complete volumes, that is, it needs to find and use a small fraction of a perfect main chain and not a complete description of the structure. This constitutes an alternative approach to the highly successful methods combining *ab initio* modelling of a nearly complete structure to be used for molecular replacement, such as RosettaMR (DiMaio *et al.*, 2011 ▶), *AMPLE* (Bibby *et al.*, 2012 ▶) or the implementation of the Zhang group (Shrestha *et al.*, 2011 ▶).

**Figure 6:**
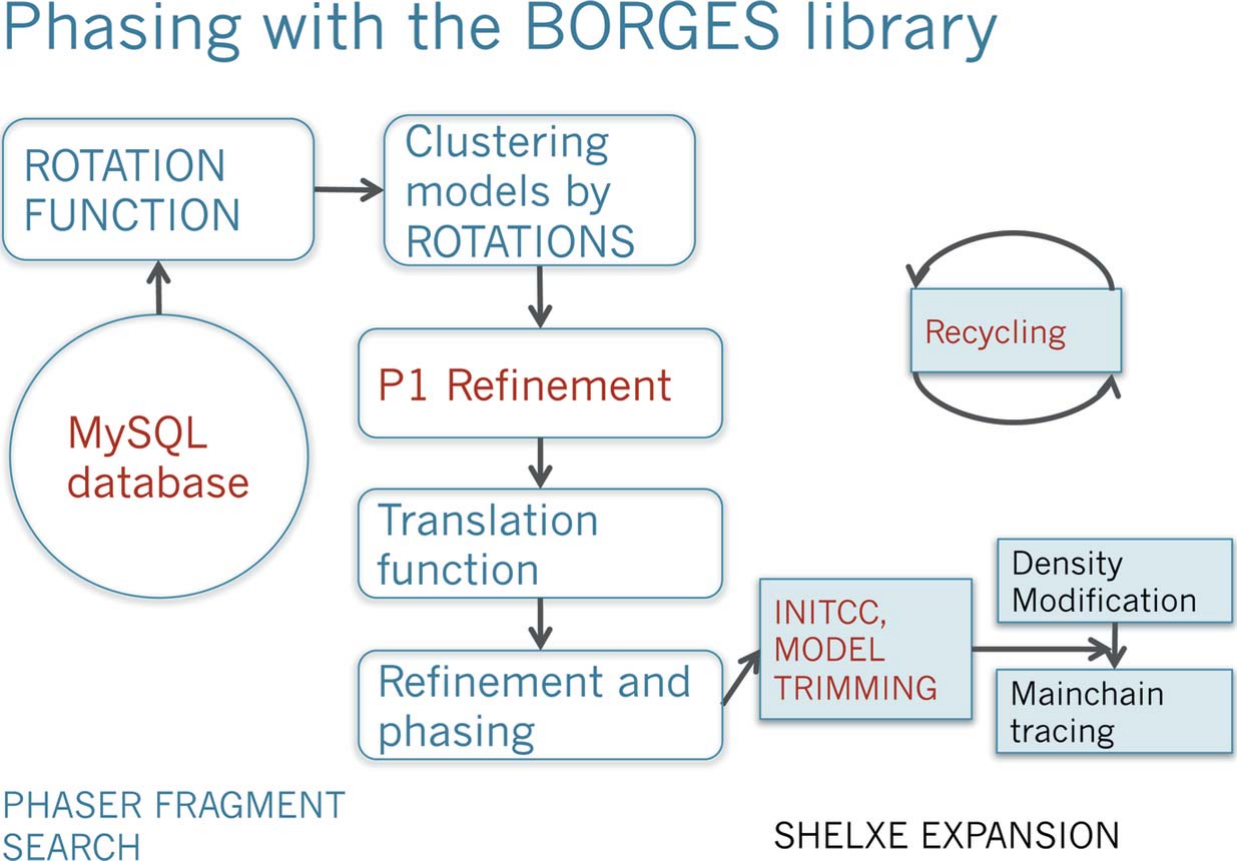
*ARCIMBOLDO_BORGES* implementation. The scheme summarizes the steps in the procedure. *PHASER* operations are printed in blue and *SHELXE* ones on a blue background. *BORGES* operations are printed in red. Starting from a model template, a library of equivalent folds is created and geometrically clustered. For each cluster, a rotation search is calculated at 2 Å. Models are dissembled and locally optimized in *P*1 with *PHASER*. Peaks are clustered geometrically, within a tolerance of 15°. Fragment location and density modification and autotracing is pursued for each model.

Exploiting unspecific local folds in an *ab initio* approach, rather than secondary structure fragments, significantly increases the dimensions of the search problem. The accuracy required is below 0.6 Å r.m.s.d. and in the absence of a hypothesis about the fold, some feeble indications can be derived from the Patterson function and secondary structure prediction but eventually libraries of the most frequent local folds have to be tried. Scoring of reduced libraries or alternative hypotheses, such as three antiparallel/parallel/parallel–antiparallel strands of the same number of amino acids is performed to establish an order. If no clear indication is provided, the most frequent case (antiparallel in this case) is attempted first. It is essential to the method, to provide internal degrees of freedom to the library models, refining them against the experimental data at two of the stages. Fig. 6 ▶ displays a scheme of the *ARCIMBOLDO_BORGES* method (http://chango.ibmb.csic.es/BORGES). To accelerate model extraction, a database is precomputed annotating the PDB structures through vectors describing main-chain geometry and useful structural features. Models extracted from this database to match a geometrical description within a given tolerance are first geometrically clustered and then clustered again through the results of a rotation function. In the phasing process, models are given internal degrees of freedom and refined against the rotation function, before proceeding on to translation search, packing filtering and rigid-group refinement. Again, model trimming to optimize the correlation coefficient is used to score hypotheses prior to density modification and autotracing. Recycling of fragment rotation and translation stages from a refined model is pursued in parallel, in order to correct possible pseudotranslated solutions (Caliandro *et al.*, 2007 ▶). The method outlined succeeds in solving the CMI structure described in §2.2, from an antiparallel three-stranded β-sheet comprising the main chain of 13 amino acids. Solution from a single perfect strand was not possible. Likewise, even all-β test structures can be solved in this way. Table 2 ▶ contains three previously unknown structures that were solved with *BORGES*.

## 5. Implementation   

The multisolution approach underlying this method requires massive computing, especially in difficult cases. Not being able to identify correct partial solutions at early stages imposes the need to forward all trials to the next stage and to try to develop them into a full solution. To complete calculations in a practical time frame, the process is split into many independent tasks and distributed over a pool of computers or a supercomputer. Our first implementation simply sent all of the calculations to a grid. Condor (Tannenbaum *et al.*, 2002 ▶) was chosen as it is ideal to manage a heterogeneous pool and it allows one to flexibly customize the use of resources, while providing robust control, ensuring every job is reallocated if one of the calculation nodes leaves the pool or a job is evicted before completion. Unfortunately, this sophisticated and powerful middleware requires more specific computing expertise and dedication to its installation and maintenance than can be usually allocated in a crystallographic laboratory. Thus, despite Condor being popular in other communities, its use is not widespread in the field of macromolecular crystallography. In the course of the last year, the program has been totally rewritten as the experience with the first implementation allowed the design of an improved algorithm. Also, it has profited from recent advances in *PHASER* (Oeffner *et al.*, 2013 ▶; Read *et al.*, 2013 ▶), allowing a much-enhanced discrimination of potentially correct partial solutions. The new version has simplified the middleware requirement and eliminated the original Condor requirement in favour of a broader choice of middleware, easier automatic access to computer pools and finally, even a version designed to run on a single machine.

### 5.1. Central implementation on a workstation with access to a pool   

**Figure 7:**
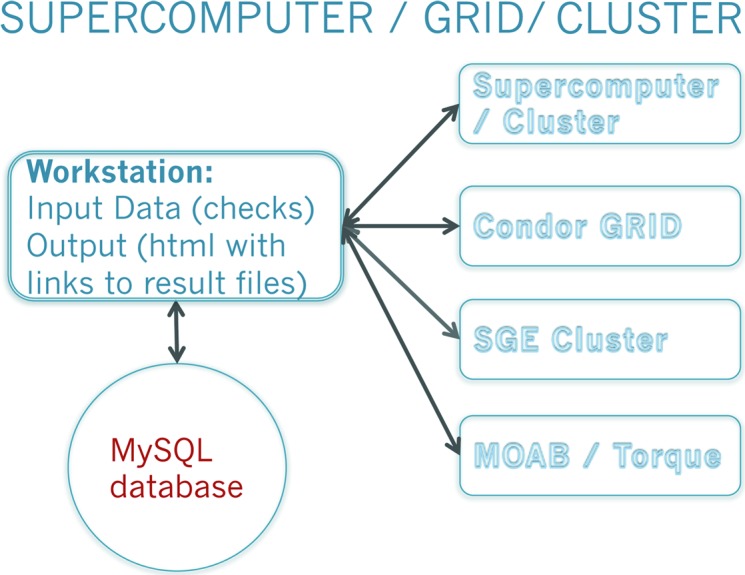
*ARCIMBOLDO–BORGES* implementation. The job is controlled from a single workstation, where output and intermediate results are accessible. For *BORGES*, a local or remote library has to be accessed, which can be shared by several users. Access to computing resources is configured providing a username and access key to the system to be exploited. The program automatically offloads heavy calculations to local or external grid pools or to a supercomputer.

The middleware dependency constituted a hurdle in the use of our method. In order to reduce it, we separated the embedded use of Condor into a more simple and flexible mode of operation, as illustrated in Fig. 7 ▶. The full implementation, designed to work with large libraries of structural models extracted from the whole PDB, relies on a database to which the workstation loads or retrieves library information. All processes central to a run take place on a single workstation, where all relevant files will be visible, so the user retains control throughout the process. The program automatically directs heavy calculations to a local or remote pool where access has been configured. In this way, access to a grid or supercomputer only needs to be configured at installation time. All the user needs is to have been granted a username and password or access key. Allocation of space and resources in the computing pool will be exploited as configured by the system manager or can be further managed in the *ARCIMBOLDO* installation to account for multiple users running under a single account on a local or remote pool. Currently, besides Condor, SGE/Opengrid (Gentzsch, 2001 ▶), Torque and MOAB are supported. In this way, the *ARCIMBOLDO* user does not need to be involved in or even perceive the supercomputing taking place. Input files, output files, interpretation and diagnostics are all visible and updated on the local workstation. An HTML page centralizes the display of results and links to the best map and trace if the structure appears to have been solved. The program depends on suitable data in mtz and hkl formats, as well as on particular versions of *PHASER* (currently 2.5.6) and *SHELXE* (latest). An initial check of these requirements may block a run if it is perceived to be doomed to failure. For instance, *ARCIMBOLDO* will not run if the resolution of the data is lower than 2.5 Å.

### 5.2. Single-machine implementation: *ARCIMBOLDO_LITE*   

**Figure 8:**
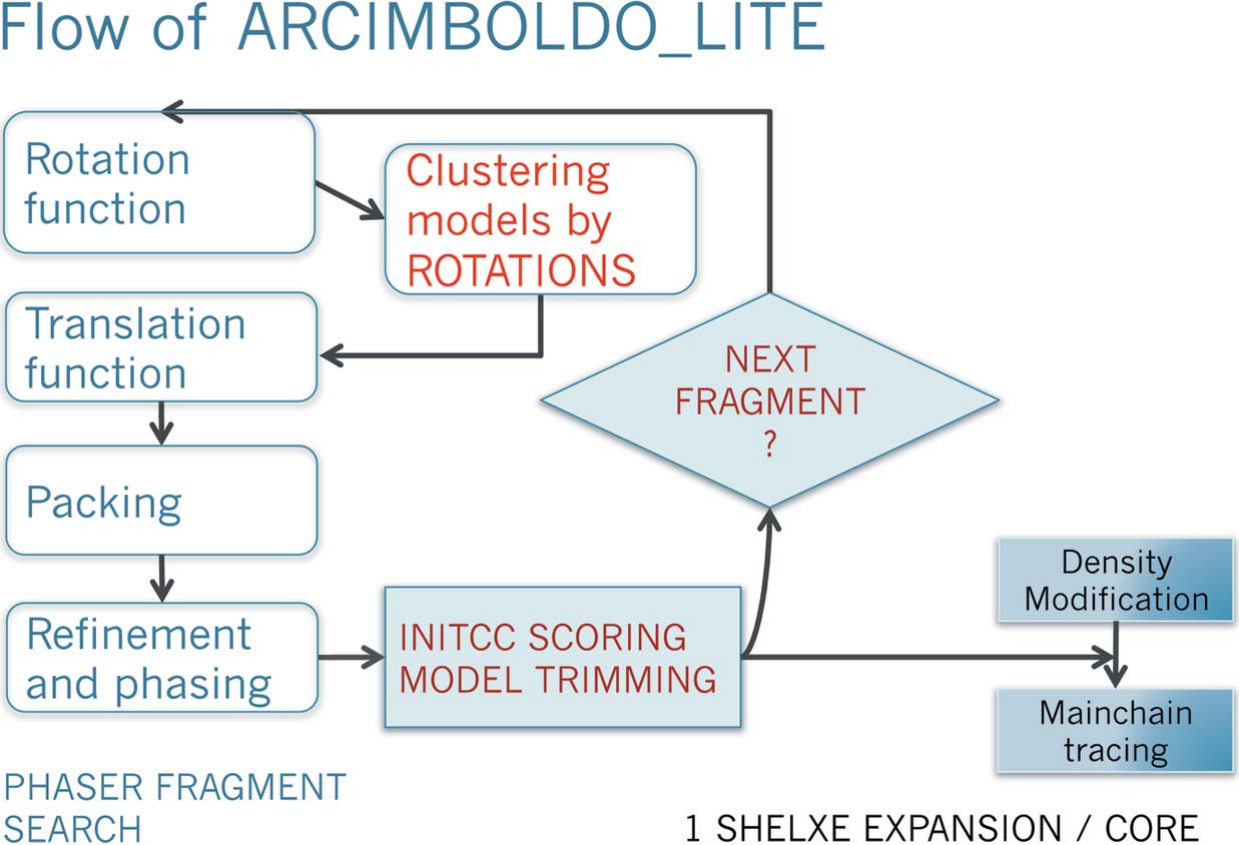
*ARCIMBOLDO_LITE* flow. From the given fragment(s), all *PHASER* operations are computed first and only as many best scoring partial solutions as available cores are expanded through density modification and autotracing.

Even the necessity of accessing a pool of computers and installing the required middleware is perceived as time consuming and user-unfriendly by many crystallographers. Analysis of past successful cases as well as test cases allowed us to propose a minimal procedure that would significantly reduce computing and consequently be able to run on a single multicore machine. The flow of *ARCIMBOLDO_LITE* is displayed in Fig. 8 ▶. The search fragment is typically a model helix of selected length that is provided internally, although any other model can be specified through a PDB file. All *PHASER* calculations are performed first and a limited number of *SHELXE* expansions will be attempted on the best scoring, not necessarily larger located substructures. The procedure is dimensioned according to the number of physical cores, therefore a run on a machine with more cores will not simply run faster, but attempt to develop more partial structures into a solution. *ARCIMBOLDO_LITE* is distributed as a single binary for Linux or MacOS and can be downloaded from http://chango.ibmb.csic.es/ARCIMBOLDO. Execution requires a single instruction file containing minimal input as suitable default values are provided for most parameters. The user needs to specify the path to the latest *PHASER* and *SHELXE* versions, the name of the diffraction data files in *SHELX* (Sheldrick, 2008 ▶) hkl and *CCP*4 (Winn *et al.*, 2011 ▶) mtz formats, the asymmetric unit composition of the target structure as well as the helix length and the number of copies to be located.

#### 5.2.1. Test structures solved with *ARCIMBOLDO_LITE*   

The structure of S100A4 in complex with nonmuscle myosin-IIA peptide (PDB ID 4eto) was used to run benchmarks for *ARCIMBOLDO_LITE* on various Linux distributions and hardware. The *P*2_1_ structure for which data to 1.54 Å are available contains 202 residues in the asymmetric unit. The structure was solved searching for four helices of 14 alanines each, taking one to two hours on machines with Debian, Ubuntu or SUSE Linux installations, with i7 or four to eight Xeon cores, a minimum of 2 GB RAM per core. A MacOS version running on the Mavericks distribution has also been tested with equivalent results.

Some of the previously reported cases of *ARCIMBOLDO* structure solution have also been reproduced with this stand­alone version. They are marked in Table 2 ▶ with an asterisk. This comprises in particular, 4e1p, 3gwh, 4k82, 4m3l, 4bjs (Shi *et al.*, 2013 ▶), and two yet unpublished structures. In addition, two previously unknown structures have first been phased with this implementation.

A set of 128 α-helix-containing structures selected from the PDB, with resolutions from 0.7 to 2.1 Å, comprising 40 to 120 amino acids in the asymmetric unit and representing all the most frequent space groups have also be solved by *ARCIMBOLDO_LITE*, on single machines with eight Intel Xeon cores and 2 GB RAM per core, in order to establish the defaults provided by the distributed program. The PDB ID codes for this set of tests are: 1a6m, 1byi, 1byz, 1c75, 1eb6, 1ejg, 1ew4, 1fk5, 1g2r, 1g2y, 1g4i, 1g6u, 1gvd, 1gxu, 1i2t, 1j8b, 1kth, 1l9l, 1n9b, 1oks, 1ox3, 1pz4, 1q8d, 1r7j, 1riy, 1rw1, 1t07, 1tgr, 1u84, 1u9p, 1uj8, 1use, 1usm, 1v2z, 1vyi, 1whz, 1wpa, 1y6x, 1yu5, 1z96, 1zva, 1zzk, 2b8i, 2c60, 2ewt, 2f60, 2fi0, 2fq3, 2fu2, 2fvy, 2g7o, 2ggc, 2gkr, 2gpi, 2h8e, 2h9u, 2hdz, 2hl7, 2hpj, 2i5f, 2igp, 2ip6, 2ivy, 2nuh, 2o1k, 2o37, 2o4t, 2o7a, 2oqq, 2ouf, 2oxo, 2p5k, 2p6v, 2pk8, 2pst, 2q2f, 2qff, 2qmt, 2qsb, 2qvo, 2r31, 2rhf, 2v75, 2vkl, 2wuj, 2zqe, 2zqm, 2zxy, 3a4c, 3bri, 3ce7, 3cec, 3cq1, 3df8, 3e21, 3e9v, 3efg, 3fbl, 3fmy, 3g21, 3g2b, 3g46, 3goe, 3h01, 3hro, 3idw, 3jsc, 3jtz, 3jvl, 3kp8, 3mxz, 3o55, 3oou, 3osh, 3qm9, 3rq9, 3s6e, 3soj, 3ui4, 3vrc, 3wh1, 3zzp, 4eic, 4g78, 4g9s, 4hs1, 4mzc, 4oo4.

Recently, a previously unknown structure with 130 residues and diffraction data to 1.5 Å has been solved using *ARCIMBOLDO_LITE* by the group led by Professor Carine Tisné at the University Descartes in Paris.

## 6. Outlook   

*Ab initio* phasing exploiting small fragments to enforce secondary and tertiary structure has allowed the solution of cases comprising several hundred amino acids in the asymmetric unit, with resolutions up to 2.1 Å, that were outside the scope of previous methods in terms of resolution limits and size. A score of previously unknown structures solved by *ARCIMBOLDO* and *BORGES* in its various modes is displayed in Table 2 ▶. Incorporation of various sources of previous knowledge into this frame allows a further relaxation of some of the limits. Use of refinement within the outlined procedures and allowing the models additional degrees of freedom increases the radius of convergence of the method. Considering all partial results jointly, rather than as isolated trials increases the efficiency and can be exploited in more economic implementations, appropriate for a single multicore machine.

## Figures and Tables

**Table 1:** Some previously unknown structures solved using *SHELXD*

Compound	Space group	No. of atoms (+ solvent)	No. of heavy atoms	Resolution ()
Actinomycin X2	*P*1	273 (305)		0.9
Actinomycin Z3	*P*2_1_2_1_2_1_	186 (307)	2 Cl	0.96
Vancomycin	*P*4_3_2_1_2	202 (312)	6 Cl	1.09
Actinomycin D	*P*1	270 (314)		0.94
Ristocetin A	*P*2_1_	294 (420)		1.03
Hirustasin	*P*4_3_2_1_2	402 (467)	10 S	1.2
Cyclodextrin	*P*2_1_	448 (467)		0.8
Decaplanin	*P*2_1_	448 (635)	4 Cl	1.00
PolyA RNA	*P*4_1_2_1_2	478 (572)	22 P	1.00
Cyclodextrin	*P*1	483 (562)		1.00
Bucandin	*C*2	516 (634)	10 S	1.05
Amylose CA26	*P*1	572 (719)	-	1.1
Viscotoxin B2	*P*2_1_2_1_2_1_	722 (812)	12 S	1.05
Mersacidin	*P*3_2_	750 (826)	24 S	1.04
Feglimycin	*P*6_5_	828 (1026)		1.10
Tsuchimycin	*P*1	1069 (1283)	24 Ca	1.00
rc-WT CvHiPIP	*P*2_1_2_1_2_1_	1264 (1599)	8 Fe	1.2
Cytochrome *c*_3_	*P*3_1_	2024 (2208)	8 Fe	1.2

**Table 2:** Previously unknown structures solved using *ARCIMBOLDO* and *BORGES* See text for information on structures marked with *.

Data from	Space group	No. ofresidues	Search fragment	Resolution ()
P. Czabotar*	*P*3_1_21	120	1H14	1.30
M. Graille (4lun)	*P*2_1_	310	1H16	1.45
C. Tisn	*P*2_1_2_1_2_1_	130	LITE 2H14	1.5
X. Gomis-Rth	*P*2_1_2_1_2_1_	560	Clustering shreds	1.5
K. v Hecke	*P*432	165	2H14	1.6
J. Hermoso	*P*6_1_	50	1H10	1.7
J. M. Pereda (4gdo)	C2	240	Composite frags 2H17	1.70
D. C. Hissa, K. Gruber (4k82)*	*P*2_1_	204	2H14	1.70
K. Zeth, A. Lupas (4gn0)	*P*2_1_	428	Composite frags 2H16	1.7
S. Trakhanov	*P*2_1_2_1_2_1_	144	1H14	1.75
V. Arcus (4e1p)*	*P*2_1_	112	2H12	1.8
CMI: K. Zeth (4aeq)*	*C*222_1_	90	1H12	1.9
T. Pavkov*	*P*4_1_2_1_2	111	1H13	1.9
M. Rudolph	*C*2	182	5H10	1.9
R. Bunker (4bjs)*	*P*1	240	Model helices	1.94
PRD2: S. Becker (3gwh)	*P*2_1_	222	3H14	1.95
HellD: Thorn, Sheldrick (3szs)	*I*422	327	2nmr31	1.95
Van Breuguel	*P*2_1_	600	Composite frags 2H17	1.97
MltE: J. Hermoso (2y8p)	*C*222_1_	378	Shredder	2.0
X. Gomis-Rth (4ija)	*P*2_1_2_1_2_1_	794	Frags + Se-MAD	2.0
Nuria Verdaguer	*P*6_3_22	50	3H14	2.1
CC-murf1: O. Mayans (4m3l)*	*P*2_1_	240	Helices with side chains	2.1
R. Hurtado	*P*2_1_2_1_2_1_	1450	Shredder	2.35
C. Artola, J. Hermoso (4c5f)	*P*2_1_	682	Frags, modelling + BUSTER	2.7
N. Valadares, R. Garratt	Pseudo-merohedral	60240	Coiled coils, twins	1.62.4

## References

[bb3] Berman, H. M., Westbrook, J., Feng, Z., Gilliland, G., Bhat, T. N., Weissig, H., Shindyalov, I. N. & Bourne, P. E. (2000). *Nucleic Acids Res.* **28**, 235–242. 10.1093/nar/28.1.235 PMC102472 10592235

[bb4] Bernstein, F. C., Koetzle, T. F., Williams, G. J. B., Meyer, E. F. Jr, Brice, M. D., Rodgers, J. R., Kennard, O., Shimanouchi, T. & Tasumi, M. (1977). *J. Mol. Biol.* **112**, 535–542. 10.1016/s0022-2836(77)80200-3 875032

[bb5] Bibby, J., Keegan, R. M., Mayans, O., Winn, M. D. & Rigden, D. J. (2012). *Acta Cryst.* D**68**, 1622–1631. 10.1107/S0907444912039194 23151627

[bb78] Bieniossek, C., Schuütz, P., Bumann, M., Limacher, A., Usón, I. & Baumann, U. (2006). *J. Mol. Biol.* **360**, 457–465. 10.1016/j.jmb.2006.05.021 16781736

[bb7] Bragg, W. H. & Bragg, W. L. (1913). *Nature*, **91**, 557.

[bb8] Bunkóczi, G., Echols, N., McCoy, A. J., Oeffner, R. D., Adams, P. D. & Read, R. J. (2013). *Acta Cryst.* D**69**, 2276–2286. 10.1107/S0907444913022750 PMC3817702 24189240

[bb9] Bunkóczi, G. & Read, R. J. (2011). *Acta Cryst.* D**67**, 303–312. 10.1107/S0907444910051218 PMC3069745 21460448

[bb10] Burla, M. C., Carrozzini, B., Cascarano, G. L., Giacovazzo, C. & Polidori, G. (2011). *J. Appl. Cryst.* **44**, 1143–1151.

[bb11] Burla, M. C., Carrozzini, B., Cascarano, G. L., Giacovazzo, C. & Polidori, G. (2012). *J. Appl. Cryst.* **45**, 1287–1294.

[bb12] Caliandro, R., Carrozzini, B., Cascarano, G. L., De Caro, L., Giacovazzo, C., Mazzone, A. & Siliqi, D. (2008). *J. Appl. Cryst.* **41**, 548–553.

[bb13] Caliandro, R., Carrozzini, B., Cascarano, G. L., De Caro, L., Giacovazzo, C. & Siliqi, D. (2005*a*). *Acta Cryst.* D**61**, 1080–1087. 10.1107/S0907444905015519 16041073

[bb14] Caliandro, R., Carrozzini, B., Cascarano, G. L., De Caro, L., Giacovazzo, C. & Siliqi, D. (2005*b*). *Acta Cryst.* D**61**, 556–565. 10.1107/S090744490500404X 15858265

[bb15] Caliandro, R., Carrozzini, B., Cascarano, G. L., De Caro, L., Giacovazzo, C. & Siliqi, D. (2007). *J. Appl. Cryst.* **40**, 883–890.

[bb16] Caliandro, R., Dibenedetto, D., Cascarano, G. L., Mazzone, A. & Nico, G. (2012). *Acta Cryst.* D**68**, 1–12. 10.1107/S0907444911046282 22194328

[bb17] DiMaio, F., Terwilliger, T. C., Read, R. J., Wlodawer, A., Oberdorfer, G., Wagner, U., Valkov, E., Alon, A., Fass, D., Axelrod, H. L., Das, D., Vorobiev, S. M., Iwaï, H., Pokkuluri, P. R. & Baker, D. (2011). *Nature*, **473**, 540–543. 10.1038/nature09964 PMC3365536 21532589

[bb19] Franke, B., Gasch, A., Rodriguez, D., Chami, M., Khan, M. M., Rudolf, R., Bibby, J., Hanashima, A., Bogomolovas, J., von Castelmur, E., Rigden, D. J., Uson, I., Labeit, S. & Mayans, O. (2014). *Open Biol.* **4**, 130172. 10.1098/rsob.130172 PMC3971405 24671946

[bb20] Frazao, C., Sieker, L., Sheldrick, G. M., Lamzin, V., LeGall, J. & Carrondo, M. A. (1999). *J. Biol. Inorg. Chem.* **4**, 162–165. 10.1007/s007750050299 10499086

[bb21] Friedrich, W., Knipping, P. & Laue, M. (1912). *Sitzungsber. K. Bayer. Akad. Wiss.* pp. 303–322.

[bb22] Fujinaga, M. & Read, R. J. (1987). *J. Appl. Cryst.* **20**, 517–521.

[bb23] Gentzsch, W. (2001). *Proc. First IEEE/ACM International Symposium on Cluster Computing and the Grid, 2001*, pp. 35–36.

[bb24] Gessler, K., Usón, I., Takaha, T., Krauss, N., Smith, S. M., Okada, S., Sheldrick, G. M. & Saenger, W. (1999). *Proc. Natl Acad. Sci. USA*, **96**, 4246–4251. 10.1073/pnas.96.8.4246 PMC16317 10200247

[bb25] Glykos, N. M. & Kokkinidis, M. (2003). *Acta Cryst.* D**59**, 709–718. 10.1107/s0907444903002889 12657790

[bb26] Gruene, T. (2013). *Acta Cryst.* D**69**, 1861–1863. 10.1107/S090744491301648X 23999309

[bb27] Hauptman, H. & Karle, J. (1953). *ACA Monograph No. 3*. Ohio: Polycrystal Book Service.

[bb28] Hendrickson, W. A. (1991). *Science*, **254**, 51–58. 10.1126/science.1925561 1925561

[bb29] Hissa, D. C., Bezerra, G. A., Birner-Gruenberger, R., Silva, L. P., Usón, I., Gruber, K. & Melo, V. M. M. (2014). *Chembiochem*, **15**, 393–398. 10.1002/cbic.201300726 24442854

[bb30] Hovmöller, S., Zhou, T. & Ohlson, T. (2002). *Acta Cryst.* D**58**, 768–776. 10.1107/s0907444902003359 11976487

[bb32] Karle, J. & Hauptman, H. (1956). *Acta Cryst.* **9**, 635–651.

[bb33] Langs, D. A. (1989). *Biopolymers*, **28**, 259–266. 10.1002/bip.360280126 2470432

[bb34] Laue, M. von (1912). *Sitzungsber. K. Bayer. Akad. Wiss.* pp. 363–373.

[bb35] Loll, P. J., Bevivino, A. E., Korty, B. D. & Axelsen, P. H. (1997). *J. Am. Chem. Soc.* **119**, 1516–1522.

[bb37] McCoy, A. J., Grosse-Kunstleve, R. W., Adams, P. D., Winn, M. D., Storoni, L. C. & Read, R. J. (2007). *J. Appl. Cryst.* **40**, 658–674. 10.1107/S0021889807021206 PMC2483472 19461840

[bb38] McCoy, A. J., Grosse-Kunstleve, R. W., Storoni, L. C. & Read, R. J. (2005). *Acta Cryst.* D**61**, 458–464. 10.1107/S0907444905001617 15805601

[bb39] McCoy, A. J., Nicholls, R. A. & Schneider, T. R. (2013). *Acta Cryst.* D**69**, 2216–2225. 10.1107/S0907444913021811 PMC3817695 24189233

[bb41] Miller, R., DeTitta, G. T., Jones, R., Langs, D. A., Weeks, C. M. & Hauptman, H. A. (1993). *Science*, **259**, 1430–1433. 10.1126/science.8451639 8451639

[bb42] Miller, R., Gallo, S. M., Khalak, H. G. & Weeks, C. M. (1994). *J. Appl. Cryst.* **27**, 613–621.

[bb44] Nascimento, A. F. Z., Trindade, D. M., Tonoli, C. C. C., de Giuseppe, P. O., Assis, L. H. P., Honorato, R. V., de Oliveira, P. S. L., Mahajan, P., Burgess-Brown, N. A., von Delft, F., Larson, R. E. & Murakami, M. T. J. (2013). *J. Biol. Chem.* **288**, 34131–34145. 10.1074/jbc.M113.507202 PMC3837155 24097982

[bb45] Nimz, O., Gessler, K., Usón, I., Sheldrick, G. M. & Saenger, W. (2004). *Carbohydr. Res.* **339**, 1427–1437. 10.1016/j.carres.2004.02.030 15178384

[bb46] Oeffner, R. D., Bunkocz, G., McCoy, A. J. & Read, R. J. (2013). *Acta Cryst.* D**69**, 2209–2215. 10.1107/S0907444913023512 PMC3817694 24189232

[bb48] Pröpper, K., Meindl, K., Sammito, M., Dittrich, B., Sheldrick, G. M., Pohl, E. & Usón, I. (2014). *Acta Cryst.* D**70**, 1743–1757. 10.1107/S1399004714007603 PMC4051508 24914984

[bb49] Ramachandran, G. N., Ramakrishnan, C. & Sasisekharan, V. (1963). *J. Mol. Biol.* **7**, 95–99. 10.1016/s0022-2836(63)80023-6 13990617

[bb50] Read, R. J., Adams, P. D. & McCoy, A. J. (2013). *Acta Cryst.* D**69**, 176–183. 10.1107/S0907444912045374 PMC3565438 23385454

[bb51] Robertson, M. P., Chi, Y.-I. & Scott, W. G. (2010). *Methods*, **52**, 168–172. 10.1016/j.ymeth.2010.06.011 PMC2948636 20541014

[bb52] Robertson, M. P. & Scott, W. G. (2008). *Acta Cryst.* D**64**, 738–744. 10.1107/S0907444908011578 PMC2507861 18566509

[bb53] Rodríguez, D. D., Grosse, C., Himmel, S., González, C., de Ilarduya, I. M., Becker, S., Sheldrick, G. M. & Usón, I. (2009). *Nat. Meth.* **6**, 651–653. 10.1038/nmeth.1365 19684596

[bb54] Rodríguez, D., Sammito, M., Meindl, K., de Ilarduya, I. M., Potratz, M., Sheldrick, G. M. & Usón, I. (2012). *Acta Cryst.* D**68**, 336–343. 10.1107/S0907444911056071 PMC3322593 22505254

[bb55] Rossmann, M. G. (1972). *The Molecular Replacement Method*. New York: Gordon and Breach.

[bb57] Sammito, M., Millán, C., Rodríguez, D. D., de Ilarduya, I. M., Meindl, K., De Marino, I., Petrillo, G., Buey, R. M., de Pereda, J. M., Zeth, K., Sheldrick, G. M. & Usón, I. (2013). *Nat. Meth.* **10**, 1099–1101. 10.1038/nmeth.2644 24037245

[bb58] Sammito, M., Meindl, K., de Ilarduya, I. M., Millán, C., Artola-Recolons, C., Hermoso J. A. & Usón, I. (2014). *FEBS J*, **281**, 4029–4045. 10.1111/febs.12897 24976038

[bb59] Safaee, N., Noronha, A. M., Rodionov, D., Kozlov, G., Wilds, C. J., Sheldrick, G. M. & Gehring, K. (2013). *Angew. Chem. Int. Ed.* **52**, 10370–10373. 10.1002/anie.201303461 23813654

[bb60] Schäfer, M., Schneider, T. R. & Sheldrick, G. M. (1996). *Structure*, **4**, 1509–1515. 10.1016/s0969-2126(96)00156-6 8994975

[bb61] Sheldrick, G. M. (2002). *Z. Kristallogr.* **217**, 644–650.

[bb62] Sheldrick, G. M. (2008). *Acta Cryst.* A**64**, 112–122. 10.1107/S0108767307043930 18156677

[bb63] Sheldrick, G. M. (2010). *Acta Cryst.* D**66**, 479–485. 10.1107/S0907444909038360 PMC2852312 20383001

[bb64] Sheldrick, G. M., Gilmore, C. J., Hauptman, H. A., Weeks, C. M., Miller, R. & Usón, I. (2011). *International Tables for Crystallography*, edited by E. Arnold, D. M. Himmel & M. G. Rossmann, pp. 413–429. Chichester: Wiley.

[bb65] Shi, T., Bunker, R. D., Mattarocci, S., Ribeyre, C., Faty, M., Gut, H., Scrima, A., Rass, U., Rubin, S. M., Shore, D. & Thomä, N. H. (2013). *Cell*, **153**, 1340–1353. 10.1016/j.cell.2013.05.007 23746845

[bb66] Shrestha, R., Berenger, F. & Zhang, K. Y. J. (2011). *Acta Cryst.* D**67**, 804–812. 10.1107/S090744491102779X 21904033

[bb67] Storoni, L. C., McCoy, A. J. & Read, R. J. (2004). *Acta Cryst.* D**60**, 432–438. 10.1107/S0907444903028956 14993666

[bb68] Tannenbaum, T., Wright, D., Miller, K. & Livny, M. (2002). *Beowulf Cluster Computing with Linux*, edited by T. Sterling. The MIT Press.

[bb69] Thorn, A. & Sheldrick, G. M. (2013). *Acta Cryst.* D**69**, 2251–2256. 10.1107/S0907444913027534 PMC3817699 24189237

[bb70] Usón, I., Patzer, S. I., Rodríguez, D. D., Braun, V. & Zeth, K. (2012). *J. Struct. Biol.* **178**, 45–53. 10.1016/j.jsb.2012.02.004 22366279

[bb71] Usón, I. & Sheldrick, G. M. (1999). *Curr. Opin. Struct. Biol.* **9**, 643–648. 10.1016/s0959-440x(99)00020-2 10508770

[bb72] Usón, I., Sheldrick, G. M., Fortelle, E. de L., Bricogne, G., Marco, S. D., Priestle, J. P., Grütter, M. G. & Mittl, P. R. E. (1999). *Structure*, **7**, 55–63. 10.1016/s0969-2126(99)80009-4 10368273

[bb74] Winn, M. D. *et al.* (2011). *Acta Cryst.* D**67**, 235–242.

[bb75] Xu, H., Hauptman, H. A., Weeks, C. M. & Miller, R. (2000). *Acta Cryst.* D**56**, 238–240. 10.1107/s0907444999014961 10666616

[bb76] Yao, J. X., Dodson, E. J., Wilson, K. S. & Woolfson, M. M. (2006). *Acta Cryst.* D**62**, 901–908. 10.1107/S0907444906008122 16855307

[bb77] Yao, J., Woolfson, M. M., Wilson, K. S. & Dodson, E. J. (2005). *Acta Cryst.* D**61**, 1465–1475. 10.1107/S090744490502576X 16239723

